# The Role of User Behaviour in Improving Cyber Security Management

**DOI:** 10.3389/fpsyg.2021.561011

**Published:** 2021-06-18

**Authors:** Ahmed A. Moustafa, Abubakar Bello, Alana Maurushat

**Affiliations:** ^1^School of Psychology, Western Sydney University, Sydney, NSW, Australia; ^2^The Marcs Institute for Brain, Behaviour and Development, Western Sydney University, Sydney, NSW, Australia; ^3^Department of Human Anatomy and Physiology, Faculty of Health Sciences, University of Johannesburg, Johannesburg, South Africa; ^4^School of Social Sciences, Western Sydney University, Sydney, NSW, Australia

**Keywords:** cyber security, social engineering, information security, phishing, cognitive hacking

## Abstract

Information security has for long time been a field of study in computer science, software engineering, and information communications technology. The term ‘information security’ has recently been replaced with the more generic term cybersecurity. The goal of this paper is to show that, in addition to computer science studies, behavioural sciences focused on user behaviour can provide key techniques to help increase cyber security and mitigate the impact of attackers’ social engineering and cognitive hacking methods (i.e., spreading false information). Accordingly, in this paper, we identify current research on psychological traits and individual differences among computer system users that explain vulnerabilities to cyber security attacks and crimes. Our review shows that computer system users possess different cognitive capabilities which determine their ability to counter information security threats. We identify gaps in the existing research and provide possible psychological methods to help computer system users comply with security policies and thus increase network and information security.

## Introduction

According to National Initiative for Cybersecurity Careers and Studies, cybersecurity is defined as ‘the activity or process, ability, or capability or state whereby information and communications systems and the information contained therein are protected from and/or defended against damage, unauthorised use or modification, or exploitation.’ Cyber and network systems involve at least four components: computer system users, security system analysts, cyber attackers, and computer systems. Cyber attackers often attempt to obtain, modify, or keep unauthorised information (Landwehr, 1981; Thompson, 2004).

Most of the research on cybersecurity has focused on improving computer network systems (Nobles, 2018), as many believe that information technology advances and software development is the main way to increase information security (Sadkhan, 2019; Benson and Mcalaney, 2020). Fewer studies have been conducted on enhancing cognitive capabilities and situational awareness of system analysts (D’Amico et al., 2005; Barford, 2010; Dutt et al., 2013; Knott et al., 2013; Tyworth et al., 2013; Mancuso et al., 2014; Gutzwiller et al., 2015; Aggarwal et al., 2018; Veksler et al., 2018).

However, cyber attackers can also manipulate the minds of computer system users, rather than a computer system itself, by, for example, using social engineering (e.g., tricking of computer system users to gain information, such as passwords) and cognitive hacking (e.g., spreading of misinformation) to break into a network or computer system (Cybenko et al., 2002; Thompson, 2004; McAlaney et al., 2015; King et al., 2018; Fraunholz et al., 2019). According to Bowen et al. (2014), social engineering attacks account for 28% of total cyber security attacks and 24% of these attacks occurred due to phishing. According to CyberEdge Reports, more than 70% of social engineering attacks have been successful in the last few years. In the 2018 and 2019 reports by Telstra, human errors are the greatest threat in cybersecurity. The reports claim that phishing (and spear-phishing) attacks were the most common attacks and they utilised partial social engineering and fraud to scam victims into installing malware or illegitimate websites to acquire their credentials. In these types of attacks, victims are often sent emails or text messages that appear, for example, to be for a software upgrade, legitimate correspondence from a third party supplier, information on a current storm or crisis, or notifications from a bank or a social networking site. In addition to falling victim to phishing attacks, computer system users also conduct other cyber security errors, such as sharing passwords with friends and family and also not installing software updates.

It is important to note that there are individual differences among computer system users in terms of complying with security behaviours. Several studies found that individual differences in procrastination, impulsivity, future thinking, and risk taking behaviours can explain differences in complying with security policies. Importantly, given the existing human errors that can impact network security, we will discuss the use of psychological methods to improve compliance with security policies. Such psychological methods include using novel polymorphic security warnings, rewarding and penalizing good and bad cyber behaviour, and increasing thinking about future consequence of actions.

This paper is structured as follows. First, we discuss studies and measures related to complying with security policies. Second, we discuss kinds of cyber security errors done by many computer system users, including falling victim to phishing, sharing passwords, and not installing software updates and. Third, we discuss individual differences underlying cyber security behaviours in computer system users, including procrastination, impulsivity, future thinking, and risk taking behaviours. We conclude by suggesting psychological methods that could be used to move user behaviour toward secure practices.

## Complying With Security Policies

Complying with security policies is one key behaviour to protect computer and network systems. There have been few studies on the psychology of compliance with security policies (Chan et al., 2005; Lee and Kozar, 2005; Hazari et al., 2009; Anderson and Agarwal, 2010; Maurushat, 2010; Guo et al., 2011). A lack of complying with security policies can significantly undermine information security (Greenwald et al., 2004; Mishra and Dhillon, 2006; West, 2008). For example, several studies have shown that computer system users often ignore security warnings (Schechter et al., 2007; Akhawe and Felt, 2013; Bravo-Lillo et al., 2013; Brase et al., 2017).

To measure such humans’ security behaviours, Egelman and Peer (2015) developed the Security Behaviour Intentions scale. The scale measures attitudes toward choosing passwords, device security, regularly updating software, and general awareness about security attacks. The scale has 16 questions, such as (a) I use a password/passcode to unlock my laptop or tablet, (b) When I’m prompted about a software update, I install it right away, (c) I manually lock my computer screen when I step away from it, and (d) If I discover a security problem, I continue what I was doing because I assume someone else will fix it. The scale itself represents very basic aspects of security protection and mitigation techniques. As we discuss below, several studies have used this scale to measure types of security errors done by computer system users.

Non-compliance with a security policy can go beyond mere ignoring warnings, choosing poor passwords or failing to adopt recommended security measures. In a recent study, Maasberg et al. (2020) found that the dark triad traits (machiavellianism, narcissism and psychopathy, machiavellianism, narcissism and psychopathy, Paulhus and Williams, 2002) correlate with malicious behaviour intentions such as insider threats. Harrison et al. (2018) recently reported that the Dark triad can explain unethical behaviour such as committing cyber fraud. The concept of Dark Triad and Big Five Methods will be explored and critiqued further in the following section.

## Human Cyber Security Errors

In this section, we describe the kinds of cyber security errors conducted by many computer system users. Several reports have shown that humans are considered the greatest vulnerability to security (Schneier, 2004; Furnell and Clarke, 2012), which has been also confirmed by recent reports. One report estimated that 95% of cyber and network attacks are due to human errors (Nobles, 2018). In our context, humans are either computer system users or security analysts (King et al., 2018; Andrade and Yoo, 2019), though most research on this area focuses on errors done by computer system users. According to Ifinedo (2014), company employees are the weakest link in ensuring system security (for discussion and analysis, also see Sasse et al., 2004; Vroom and von Solms, 2004; Stanton et al., 2005; Guo et al., 2011).

Some human errors related to cyber and network security include, but not limited to, sharing passwords, oversharing information on social media, accessing suspicious websites, using unauthorised external media, indiscriminate clicking on links, reusing the same passwords in multiple places, opening an attachment from an untrusted source, sending sensitive information via mobile networks, not physically securing personal electronic devices, and not updating software (Boyce et al., 2011; Calic et al., 2016). Along these lines, one main issue underlying information and cyber security is the dilemma of increasing availability and ease to access a network or data but, at the same time, maintain security (Veksler et al., 2018). To increase security, organisations often require computer system users to have complex passwords, which makes usability quite difficult. Computer system users, however, tend to take the path of least resistance, such as using a weak password and using the same password for several websites. Below, we discuss prior studies on three kinds of human security errors: falling victim to phishing, sharing passwords with others, and installing software updates.

**Falling victim to phishing:** Some phishing studies have used a laboratory-based phishing experiment (Jakobsson and Ratkiewicz, 2006; Jagatic et al., 2007). The use of laboratory-based phishing experiment has been shown in a recent study to relate to real-life phishing (Hakim et al., 2020). One study found that over 30% of government employees click on a suspicious link in this phishing email, and many of these have provided their passwords (Baillon et al., 2019). In another study using a similar phishing experiment, around 60% of university students clicked on suspicious link in a phishing email (Diaz et al., 2018). Accordingly, several studies suggest that human factors, behavioural studies, and psychological research must be considered in cyber and network security studies (Hamill and Deckro, 2005; Jones and Colwill, 2008). In another study, Bowen et al. (2014) studied how Columbia University students and academic staff respond to phishing emails, and found that it took people around 4 rounds to discover they are receiving phishing emails.

One recent study also found that a successful phishing attack is related to the Dark Triad traits of the computer users, including machiavellianism, narcissism, and psychopathy (Curtis et al., 2018). In this study, it was found that high scores in narcissism is related to a higher tendency to fall victim to phishing attempts. Along these lines, it was found that neuroticism is related to falling victim to phishing attacks (Halevi et al., 2013). In another study by Gonzalez and colleagues (Rajivan and Gonzalez, 2018), it was found that the use of some cyberattack strategies, such as sending excessive amount of notification and expressing shared interest, were more related to successful phishing.

One study found that even warning people about phishing does not change their response to phishing emails (Mohebzada et al., 2012). Using the Human Aspects of Information Security Questionnaire (HAIS-Q) (Calic et al., 2016; Parsons et al., 2017), it was found that individuals who scored high on the HAIS-Q performed better on a laboratory-based phishing experiment, in which a randomly selected sample of participants (from a firm, university, school, or so) are unknowingly sent a phishing email that urges them to share their password. Herath and Rao (2009) found that computer system users generally underestimate the probability of security breaches and cybercrimes happening to them.

**Sharing passwords:** Sharing passwords with friends and family, and even strangers is a prevalent example of human cyber security errors. According to Whitty et al. (2015), older adults who score high on perseverance and self-monitoring are more likely to share passwords. Sharing passwords may lead to financial exploitation of older adults, which is among the most common forms of abuse (Bailey et al., 2015). This is the case as many older adults are very trusting of others and strangers, especially on the internet. Like older adults, younger adults also share passwords, especially ones for streaming systems. Younger users (who had grown up with computers) perceived security as an obstacle they had to work around (Smith, 2003). Sharing passwords is generally problematic as most people often use the same passwords for several websites, and thus by sharing a password, others can access their other secure information. One problem with using the same password in many systems is that cybercriminals, once find these passwords in one system, can use these passwords in many other websites.

**Installing software updates:** One common error underlying cybersecurity behaviours is a delay in or even not at all installing software updates (Rajivan et al., 2020). Using an experimental behavioural decision making study, Rajivan et al. (2020) found that risk-taking behaviours can partly explain some individuals behaviours regarding installing software updates, such that individuals who are more risk taking tend to delay the installation of software updates. Unlike sharing passwords and phishing, the area of installing software updates has not received much attention in the field.

## Individual Differences Underlying Cyber Security Behaviours

Individual differences in personality, cognitive and behavioural traits are related to cyber security behaviours. Dawson and Thomson (2018) argue that individual differences in cognitive abilities and personality traits can play a key role in success to secure computer and information systems. Below, we discuss some of these psychological traits.

**Procrastination:** Complying with security policies is possibly related to cognitive processes, such as working hard to achieve certain goals. One scale, known as “the need for cognition” scale measures working hard, enjoying and participating in activities that require efforts and thinking (Lin et al., 2016). Along these lines, Egelman and Peer (2015) found that performance in the Security Behaviour Intentions Scale is related to the Need for Cognition (NFC), which refers to inclination to exerting cognitive efforts (Cacioppo et al., 1984). Interestingly, a new study has developed a scale to measure procrastination in children and adolescents, which is suitable for the increasing number of young internet users (Keller et al., 2019). Along these lines, Shropshire et al. (2006) reported a link between the intent to comply with information security protocols and conscientiousness (i.e., doing work thoroughly and accurately) (McBride et al., 2012). Further, using the General Decision-Making Style (GDMS) scale (Scott and Bruce, 1995), Egelman and Peer (2015) found that performance in the Security Behaviour Intentions Scale is related to procrastination, such that, individuals who procrastinate were less likely to follow security policies. This is plausible as procrastination is negatively correlated with active participation in activities (Sarmany-Schuller, 1999).

**Impulsivity:** Complying with security policies may be also related to individual differences in impulsive behaviours. Egelman and Peer (2015) found that performance in the Security Behaviour Intentions Scale is related to Barratt Impulsiveness Scale scores (Patton et al., 1995). Another study found that internet addiction and impulsivity predicts risky cyber behaviours (Hadlington, 2017). Along these lines, Hu et al. (2015) found that individual differences in self and cognitive control (a key feature of impulsive behaviours) is related to violation of information security policies. Wiederhold (2014) also found that people fall victim to cybersecurity attacks in the pursuit of immediate gratification. One key feature related to impulsivity is thinking about future consequences of one’s actions (e.g., saving money now to buy a house in the future vs. spending all money now to enjoy life).

**Future thinking:** Importantly, complying with security policies may also be related to thinking about the future as well as impact of present actions on future consequences (A. A. Moustafa et al., 2018a; Moustafa et al., 2018b). In other words, individuals who think more about the future may abide by security rules to make sure their computer system is safe in the future. Along these lines, Egelman and Peer (2015) found that performance in the Security Behaviour Intentions Scale is related to Consideration for Future Consequences (CFC) (Joireman et al., 2012). This scale includes items that are very relevant to cyber security behaviours, such as ‘I consider how things might be in the future, and try to influence those things with my day to day behaviour’, ‘I think it is important to take warnings about negative outcomes seriously even if the negative outcome will not occur for many years’, and ‘When I make a decision, and I think about how it might affect me in the future’.

**Risk taking behaviours:** Another personality trait related to cyber security is risk taking behaviours. Some studies have found that computer system users who are high in risk taking may be more likely to fall victims to cybercrimes (Henshel et al., 2015; King et al., 2018). Risk is defined as engaging in a behaviour with an uncertain outcome, usually for the benefit of gaining more (Saleme et al., 2018). For example, robbing a bank is risky, as one may get caught. A lack of complying with security policies is risky as the benefit is not doing any additional work, such as software update (which is rewarding), but the risk is falling victim to cybercrimes and phishing. Another example is finding out that there has been a data breach where your personal information such as your username and password has been compromised, but then not doing anything to change your password. The dilemma computer system users face is doing additional work to secure their network or computer systems (too much work but more safe) or not (less work but less safe). Importantly, Egelman and Peer (2015) found that performance in the Security Behaviour Intentions Scale is related to performance in the Domain-Specific Risk-Taking Scale, which has items on general risk taking behaviours in everyday life (Blais and Weber, 2006; Saleme et al., 2018; Saleme and Moustafa, 2020). In several studies, by using the Risky Cybersecurity Behaviours Scale, Security Behaviours Intentions Scale (SeBIS), and Attitudes toward cybersecurity and cybercrime in business (ATC-IB), Hadlington and colleagues (Hadlington, 2017; Hadlington and Murphy, 2018) found that heavy media multitasking is associated with risky cybersecurity behaviours and increased cognitive errors.

Optimism bias is related to risk-based decision making. There have few psychology studies on optimism bias in humans (West, 2008; Sharot, 2011; Moutsiana et al., 2013; Garrett and Sharot, 2017). Generally, people assume that the best will happen to them, and they do not think they are at risk (West, 2008), that is, humans tend to be more optimistic and discount the likelihood of negative events happening to them. For example, people generally do not assume they will have cancer disease, and often discount the likelihood of it happening. This is relevant to research on the psychology of cyber and network security as computer system users may tend to discount the impact of cyber-attacks or crimes happening to them. For example, one study found that people fall victim to cybersecurity attacks due to optimism bias (Wiederhold, 2014). Importantly, future work should investigate individual differences in optimism bias and its relationship to risky cybersecurity behaviours.

Other areas of study that have examined individual differences in cybersecurity are considered under the framework of the Dark Triad and the Big Five Model. The majority of these studies are in the field of cyber bullying which falls outside of the scope of this paper, but other studies have been incorporated into sections of this paper (West, 2008; Goodboy and Martin, 2015; Jacobs et al., 2015; Alonso and Romero, 2017; Rodriguez-Enriquez et al., 2019; Curtis et al., 2021). The Big Five Scale has also been used in cybersecurity and psychology studies. The Big Five Scales refers to Agreeableness, Neuroticism, Openness, Conscientious and Extraversion. We have found, however, that the literature refers to only Neuroticism, Openness and Extraversion. Instead of examining the individual differences of the limited approach of the dark triad and the Big Five Scales we have instead pulled out the multi-dimensional aspects involved with the triad. For example, impulsivity is one component that expands across the different indexes of measurement. The other factors are grouped in Table 1.

**TABLE 1:** Summary of individual traits founds in applicable theories and instruments.

**Individual trait**	**Test/theory**	**Instrument**
Procrastination	Big Five:	Hunter and Schmidt Meta-Analysis Procedure
	Neuroticism	Academic Procrastination Scale
	Dark Triad:	Adult Inventory of Procrastination
	Machiavellianism	Aitken Procrastination Inventory
	and Psychopathy	Decisional Procrastination Questionnaires
		General Procrastination Scale
		Procrastination Assessment Scale—Students
		Procrastination Log—Behaviour
		Procrastination Self-Statement Inventory
		Test Procrastination Questionnaire
Impulsiveness	Dark Triad:	Hadlington’s Examination
	Psychopathy	Abbreviated Impulsiveness Scale
	Narcissism	Barratt’s Impulsiveness Scale
	Big 5 Scales:	Security Behaviours Intentions Scale (SeBIS)
	Openness	Ecological Momentary Assessment
	Extraversion	Dysfunctional Impulsivity subscale of the Dickman
		Impulsivity Inventory
Future thinking		Internet Addiction Test
		Wishful Thinking Scale
		Automatic Thoughts Questionnaire
		Entrepreneurial Self-Efficacy (ESE) scale
		Cyber Bullying Attitude Scale
		Cybersecurity Attitudes Scale
Risk taking		Security Behaviour Intentions Scale
		Domain Specific Risk Taking Scale
		Risky Cybersecurity Behaviours Scale

In sum, in this section, we reviewed prior studies showing that personality traits and individual differences in procrastination, impulsivity, and risk-taking behaviours, are related to cyber security behaviours.

## Improving Security Behaviours Using Psychological Methods

As discussed above, cyber attackers often use social engineering and cognitive hacking methods to break into a network or computer systems (Cybenko et al., 2002; Thompson, 2004; McAlaney et al., 2015; King et al., 2018; Fraunholz et al., 2019). Some computer system users may have some personality traits that make them likely to fall victims to phishing. Accordingly, it is important to equip vulnerable computer system users (i.e., those who may not comply with security policies) with capabilities to mitigate these effects. In this section, we discuss several psychological methods to increase compliance with security policies.

**Using novel polymorphic security warnings:** According to Anderson et al. (2015), most people ignore security warnings on the internet due to habituation. In the field of psychology, habituation refers to a decreased response to repeated exposure to the same stimulus over time (Rankin et al., 2009). That is, we do not pay attention to objects that we repeatedly see. West (2008) also argued that most warning messages are similar to other message dialogs. Accordingly, computer system users often ignore them, as our brain is not likely to show novelty and attentional allocation response to such security warnings (Moustafa et al., 2009).

According to Wogalter (2006), the use of different polymorphic security warnings over time will help increase attention to these warnings. Along these lines, Anderson et al. (2015) found that the use of polymorphic warnings did not lead to habituation, that is, computer system users can still pay attention and respond to these security warnings. Similar findings were also found by Brustoloni and Villamarín-Salomón (2007). Responding to novel and anomalous activities are aspects of situational awareness, and key for detecting phishing attempts in a cyber or network systems (D’Amico et al., 2005; Barford, 2010; Dutt et al., 2013; Knott et al., 2013; Tyworth et al., 2013; Mancuso et al., 2014; Aggarwal et al., 2018; Veksler et al., 2018). Software engineers should develop attention-capturing security warnings and not standard message dialogs, and these also should change over time in order to increase alertness and attention in computer system users. Using unique and novel security messages is important, as research have reported that these messages can increase brain activation and attentional processes (Moustafa et al., 2009, 2010; Kar et al., 2010).

In addition, other studies have compared security warning design differences between Firefox, Google and Internet Explorer browsers (Akhawe and Felt, 2013). Akhawe and Felt found that browser security warnings can be effective security mechanisms although there were a number of important variables that contribute to click through rates after warnings including warning type, number of clicks, warning appearance, certificate pinning and time spent on warnings.

**Rewarding and penalizing good and bad cyber behaviour:** In everyday life, we learn from negative (e.g., loss, penalties, etc.) or positive (e.g., reward) outcomes. Humans are often motivated to do certain actions to receive reward and avoid negative outcomes (Frank et al., 2007; Moustafa et al., 2008, 2013, 2015, 2017; Bodi et al., 2009; Piray et al., 2014; Myers et al., 2016). However, in the case of cyber security behaviours, the reward is that nothing bad will happen; that is, the user’s computer system will not be attacked if they comply with security policies. In other words, complying with cyber security behaviours is an example of negative reinforcement in which actions (i.e., complying with cyber security policies) prevent the occurrence of a negative outcome (Sidman, 2006; May et al., 2020).

Based on these findings, the use of more concrete rewards and losses may increase compliance with security policies. For example, companies should enforce fines (kind of punishment learning) on employees who do not adhere to security policies and reward ones who do. Maqbool et al. (2020) argued that penalizing individuals should increase security behaviours. Along these lines, Baillon et al. (2019) used a phishing experiment (in which participants click on a link which then ask them to provide their passwords) to study how simulated experience with prior phishing can impact future behaviour. They found that experiencing simulated phishing (i.e., a negative outcome) increases compliance with security policies in the computer system users. It has been found that providing information about the prevalence of phishing (i.e., negative outcome can occur to people) can decrease clicking on suspicious links in phishing emails (Baillon et al., 2019). Accordingly, computer system users should be provided with simulated experience of negative outcomes that may occur due to their erroneous cyber security policies. Further, future studies should explore whether rewarding compliance with security policies will increase future pro security behaviours (Regier and Redish, 2015).

Along these lines, according to Tversky and Kahneman (1986), most people prefer a certain small reward over uncertain big reward, but people prefer uncertain loss than a certain loss (for discussion, also see for discussion, also see Herzallah et al., 2013). In other words, people generally prefer to gamble on losses. This is evident in security behaviours. Given that the reward related to security behaviours is not direct (i.e., nothing bad will happen), using a strong reward should increase adherence to security behaviours. Future research should also investigate the relationship between individual differences in response to rewarding and penalizing outcomes and compliance with security behaviours.

**Increasing thinking about future consequence of actions:** As mentioned above, some of the key features about lack of complying with cyber security policies is not thinking much about future consequences. It has been found that thinking about future consequences is related to reflective decision making and planning (Eskritt et al., 2014) and can decrease impulsive behaviours, which is related to risky behaviours on the web as we discussed above (Bromberg et al., 2015, 2017). Accordingly, using psychological methods to increase thinking about future consequences of actions can help increase reflective decision making, and thus improve cyber security behaviours (Altintas et al., 2020).

## Conclusion and Future Directions

Our review shows that some personality traits, such as impulsivity, risk taking, and lack of thinking about future consequences of actions, are related to a lack of compliance with cyber and network security policies. Future research should focus on developing a battery of tests to integrate personality traits and cognitive processes related to cyber and network security behaviours in one framework. This battery of tests should include cognitive processes discussed above, including impulsivity, risk taking, and thinking about future consequences of actions. Furthermore, here, we show that some psychological methods can increase pro-security behaviours, such as rewarding and penalizing security-related behaviours, using novel polymorphic security warnings, and using psychological methods to increase thinking about future consequences of actions. In addition, there are cognitive training methods, including working memory training, that help reduce impulsivity, risk taking and procrastination in the general population (Rosenbaum et al., 2017; Peckham and Johnson, 2018). Such cognitive training methods can be used to ameliorate these behavioural traits and help improve cybersecurity behaviours.

As discussed above, there are different kinds of human errors that can undermine computer and security systems, including sharing passwords, oversharing information on social media, accessing suspicious websites, using unauthorised external media, indiscriminate clicking on links, reusing the same passwords in multiple places, using weak passwords, opening an attachment from an untrusted source, sending sensitive information via mobile networks, not physically securing personal electronic devices, and not updating software. However, most of the research conducted on human errors has been on phishing emails and sharing passwords. Future research should also investigate individual differences and contextual information (e.g., mood status, urgency at work, or multitasking) underlying other kinds of cyber security errors, such as using same or weak passwords in several websites, not connecting with virtual private networks and not encrypting data.

There are computational cognitive models applied to cybersecurity (for a review, see Veksler et al., 2018; Veksler et al., 2020). Veksler et al. (2020) argue that such cognitive models can used to predict the behaviour of attackers or computer system users. For example, Sandouka et al. (2009) used neural network models to detect social engineering attacks. The model was applied to phone conversation data, which include logs of phone calls. Each log includes date, time, where the call originated and terminated, and details of the conversation (Hoeschele, 2006). The model was used to analyse the text and detect any intrusions or social engineering attempts. Furthermore, Maqbool et al. (2020) used cognitive modeling and found that an excessive reliance on recency and frequency are related to cyber-attacks. However, future work should use computational models to better understand the relationship between cognitive processes and cybersecurity behaviours.

## Author Contributions

All authors listed have made a substantial, direct and intellectual contribution to the work, and approved it for publication.

## Conflict of Interest

The authors declare that the research was conducted in the absence of any commercial or financial relationships that could be construed as a potential conflict of interest.

## References

[B1] Aggarwal P. Frédéric M. Gonzalez M. C. Dutt V. (2018). Understanding cyber situational awareness in a cyber security game involving recommendation. *Int. J. Cyber Situat. Aware.* 3 11–38. 10.22619/ijcsa.2018.100118

[B2] Akhawe D. Felt A. P. (2013). “Alice in warningland: a large-scale field study of browser security warning effectiveness,” in *Proceedings of the 22nd USENIX Security Symposium*, Washington, DC.

[B3] Alonso C. Romero E. (2017). Aggressors and victims in bullying and cyberbullying: a study of personality profiles using the five-factor model. *Span. J. Psychol.* 20:e76. 29199631 10.1017/sjp.2017.73

[B4] Altintas E. Karaca Y. Moustafa A. A. El Haj M. (2020). Effect of best possible self intervention on situational motivation and commitment in academic context. *Learn. Motiv.* 69:101599. 10.1016/j.lmot.2019.101599

[B5] Anderson B. B. Kirwan C. B. Jenkins J. L. Eargle D. (2015). “How polymorphic warnings reduce habituation in the brain—insights from an fmri study,” in *Proceedings of the 33rd Annual ACM Conference on Human Factors in Computing Systems CHI, Crossings*, Seoul.

[B6] Anderson C. L. Agarwal R. (2010). Practicing safe computing: a multimethod empirical examination of home computer user security behavioral intentions. *MIS Q.* 34 613–643. 10.2307/25750694

[B7] Andrade R. O. Yoo S. G. (2019). Cognitive security: a comprehensive study of cognitive science in cybersecurity. *J. Inf. Secur. Appl.* 48:102352. 10.1016/j.jisa.2019.06.008

[B8] Bailey P. E. Slessor G. Rieger M. Rendell P. G. Moustafa A. A. Ruffman T. (2015). Trust and trustworthiness in young and older adults. *Psychol. Aging* 30 977–986. 10.1037/a0039736 26389525

[B9] Baillon A. de Bruin J. Emirmahmutoglu A. van de Veer E. van Dijk B. (2019). Informing, simulating experience, or both: a field experiment on phishing risks. *PLoS One* 14:e0224216. 10.1371/journal.pone.0224216 31851688 PMC6919577

[B10] Barford P. (2010). “Cyber SA: situational awareness for cyber defense,” in *Cyber Situational Awareness.* Advances in Information Security Vol. 46 eds Liu P. Jajodia S. Swarup V. Wang C. (Boston, MA: Springer).

[B11] Benson V. Mcalaney J. (2020). *Cyber Influence and Cognitive Threats.* Cambridge, MA: Academic Press.

[B12] Blais A. R. Weber E. U. (2006). A domain-specific risk-taking (dospert) scale for adult populations. *Judgm. Decis. Mak.* 1 33–47.

[B13] Bodi N. Keri S. Nagy H. Moustafa A. Myers C. E. Daw N. (2009). Reward-learning and the novelty-seeking personality: a between- and within-subjects study of the effects of dopamine agonists on young Parkinson’s patients. *Brain* 132 (Pt 9) 2385–2395. 10.1093/brain/awp094 19416950 PMC2766178

[B14] Bowen B. M. Devarajan R. Stolfo S. (2014). “Measuring the human factor of cyber security,” in *Proceedings of the 2011 IEEE International Conference on Technologies for Homeland Security (HST)*, Waltham, MA.

[B15] Boyce M. W. Duma K. M. Hettinger L. J. Malone T. B. Wilson D. P. Lockett-Reynolds J. (2011). “Human performance in cybersecurity: a research agenda” in *Proceedings of the Human Factors and Ergonomics Society 55th Annual Meeting.*

[B16] Brase G. L. Vasserman E. Y. Hsu W. (2017). Do different mental models influence cybersecurity behavior? Evaluations via statistical reasoning performance. *Front. Psychol.* 8:1929. 10.3389/fpsyg.2017.01929 29163304 PMC5673648

[B17] Bravo-Lillo C. Komanduri S. Cranor L. Reeder R. Sleeper M. Downs J. (2013). “Your attention please: designing security-decision UIs to make genuine risks harder to ignore,” in *Proceedings of the Symposium on Usable Privacy and Security (SOUPS)*, Newcastle.

[B18] Bromberg U. Lobatcheva M. Peters J. (2017). Episodic future thinking reduces temporal discounting in healthy adolescents. *PLoS One* 12:e0188079. 10.1371/journal.pone.0188079 29166658 PMC5699809

[B19] Bromberg U. Wiehler A. Peters J. (2015). Episodic future thinking is related to impulsive decision making in healthy adolescents. *Child. Dev.* 86 1458–1468. 10.1111/cdev.12390 26110500

[B20] Brustoloni J. C. Villamarín-Salomón R. (2007). “Improving security decisions with polymorphic and audited dialogs,” in *Proceedings of the SOUPS’07: 3rd Symposium on Usable Privacy and Security* New York, NY, 76–85. 10.1145/1280680.1280691

[B21] Cacioppo J. T. Petty R. E. Feng Kao C. (1984). The efficient assessment of need for cognition. *J. Pers. Assess.* 48 306–307. 10.1207/s15327752jpa4803_13 16367530

[B22] Calic D. Pattinson M. Parsons K. (2016). “Naive and accidental behaviours that compromise information security: what the experts think,” in *Proceedings of the 10th International Symposium of Human Aspects of Information Security and Assurance*, eds Clarke N. L. Furnell S. M. (Frankfurt: HAISA).

[B23] Chan M. Woon I. M. Y. Kankanhalli A. (2005). Perceptions of information security at the workplace: linking information security climate to compliant behavior. *J. Inf. Privacy Secur.* 1 18–41. 10.1080/15536548.2005.10855772

[B24] Curtis S. Basak A. Carre J. Bošanskı B. Èernı J. Ben-Asher N. (2021). The Dark Triad and strategic resource control in a competitive computer game. *Pers. Individ. Diff.* 168:110343. 10.1016/j.paid.2020.110343

[B25] Curtis S. R. Rajivan P. Jones D. N. Gonzalez C. (2018). Phishing attempts among the dark triad: patterns of attack and vulnerability. *Comput. Hum. Behav.* 87 174–182. 10.1016/j.chb.2018.05.037

[B26] Cybenko G. Giani A. Thompson P. (2002). Cognitive hacking: a battle for the mind. *Computer* 35 50–56. 10.1109/mc.2002.1023788

[B27] D’Amico A. Whitley K. Tesone D. (2005). “Achieving cyber defense situational awareness: a cognitive task analysis of information assurance analysts,” in *Proceedings of the Human Factors and Ergonomics Society Annual Meeting*, Los Angeles, CA.

[B28] Dawson J. Thomson R. (2018). The future cybersecurity workforce: going beyond technical skills for successful cyber performance. *Front. Psychol.* 9:744. 10.3389/fpsyg.2018.00744 29946276 PMC6005833

[B29] Diaz A. Sherman A. T. Joshi A. (2018). Phishing in an academic community: a study of user susceptibility and behavior. *arXiv* [Preprint] arXiv:1811.06078

[B30] Dutt V. Ahn Y. Gonzalez C. (2013). Cyber situation awareness: modeling detection of cyber attacks with instance-based learning theory. *Hum. Factors* 55 605–618. 10.1177/0018720812464045 23829034

[B31] Egelman S. Peer E. (2015). Scaling the security wall developing a security behavior intentions scale (SEBIS). *Paper Presented at the Security Feedback & Warnings CHI*, Seoul.

[B32] Eskritt M. Doucette J. Robitaille L. (2014). Does future-oriented thinking predict adolescent decision making? *J. Genet. Psychol.* 175 163–179. 10.1080/00221325.2013.875886 24796162

[B33] Frank M. J. Moustafa A. A. Haughey H. M. Curran T. Hutchison K. E. (2007). Genetic triple dissociation reveals multiple roles for dopamine in reinforcement learning. *Proc. Natl. Acad. Sci. U.S.A.* 104 16311–16316. 10.1073/pnas.0706111104 17913879 PMC2042203

[B34] Fraunholz D. Anton S. D. Lipps C. Reti D. Krohmer D. Pohl F. (2019). Demystifying deception technology: a survey. *arXiv* [Preprint] arXiv:1804.06196

[B35] Furnell S. Clarke C. (2012). Power to the people? The evolving recognition of human aspects of security. *Comput. Secur.* 31 983–988. 10.1016/j.cose.2012.08.004

[B36] Garrett N. Sharot T. (2017). Optimistic update bias holds firm: three tests of robustness following Shah et al. *Conscious Cogn.* 50 12–22. 10.1016/j.concog.2016.10.013 27836628 PMC5380127

[B37] Goodboy A. Martin M. (2015). The personality profile of a cyberbully: examining the dark triad. *Comput. Hum. Behav.* 49 1–4. 10.1016/j.chb.2015.02.052

[B38] Greenwald S. J. Olthoff K. G. Raskin V. Ruch W. (2004). The user non-acceptance paradigm: INFOSEC’s dirty little secret. *Paper Presented at the New Security Paradigms Workshop*, New York, NY.

[B39] Guo K. H. Yuan Y. Archer N. P. Connelly C. E. (2011). Understanding nonmalicious security violations in the workplace: a composite behavior model. *J. Manag. Inf. Syst.* 28 203–236. 10.2753/mis0742-1222280208

[B40] Gutzwiller R. S. Fugate S. Sawyer B. D. Hancock P. A. (2015). The human factors of cyber network defense. *Paper presented at the In Proceedings of the Human Factors and Ergonomics Society Annual Meeting*, Los Angeles, CA.

[B41] Hadlington L. (2017). Human factors in cybersecurity; examining the link between Internet addiction, impulsivity, attitudes towards cybersecurity, and risky cybersecurity behaviours. *Heliyon* 3:e00346. 10.1016/j.heliyon.2017.e00346 28725870 PMC5501883

[B42] Hadlington L. Murphy K. (2018). Is media multitasking good for cybersecurity? exploring the relationship between media multitasking and everyday cognitive failures on self-reported risky cybersecurity behaviors. *Cyberpsychol. Behav. Soc. Netw.* 21 168–172. 10.1089/cyber.2017.0524 29638157 PMC5882175

[B43] Hakim Z. M. Ebner N. C. Oliveira D. S. Getz S. J. Levin B. E. Lin T. (2020). The phishing email suspicion test (PEST) a lab-based task for evaluating the cognitive mechanisms of phishing detection. *Behav. Res. Methods* 10.3758/s13428-020-01495-0 [Epub ahead of print]. 33078362 PMC8188181

[B44] Halevi T. Lewis J. Memon N. (2013). “A pilot study of cyber security and privacy related behaviour and personality traits,” in *Proceedings of the WWW’13 Companion: 22nd International Conference on World Wide Web* Rio de Janeiro.

[B45] Hamill R. F. Deckro J. M. K. (2005). Evaluating information assurance strategies. *Decis. Support Syst.* 39 463–484. 10.1016/j.dss.2003.11.004

[B46] Harrison A. Summers J. Mennecke B. (2018). The effects of the dark triad on unethical behavior. *J. Bus. Ethics* 153 53–77. 10.1007/s10551-016-3368-3

[B47] Hazari S. Hargrave W. Clenney B. (2009). An empirical investigation of factors influencing information security behavior. *J. Inf. Privacy Secur.* 4 3–20. 10.1080/2333696x.2008.10855849

[B48] Henshel D. Cains M. G. Hoffman B. Kelley T. (2015). Trust as a human factor in holistic cyber security risk assessment. *Proc. Manuf.* 3 1117–1124. 10.1016/j.promfg.2015.07.186

[B49] Herath T. Rao H. R. (2009). Protection motivation and deterrence: a framework for security policy compliance in organisations. *Eur. J. Inf. Syst.* 18 106–125. 10.1057/ejis.2009.6

[B50] Herzallah M. M. Moustafa A. A. Natsheh J. Y. Abdellatif S. M. Taha M. B. Tayem Y. I. (2013). Learning from negative feedback in patients with major depressive disorder is attenuated by SSRI antidepressants. *Front. Integr. Neurosci.* 7:67. 10.3389/fnint.2013.00067 24065894 PMC3779792

[B51] Hoeschele M. (2006). *Detecting Social Engineering.* CERIAS Tech Report 2006-15. Ph.D. Thesis. West Lafayette, IN: Purdue University.

[B52] Hu Q. West R. Smarandescu L. (2015). The role of self-control in information security violations: insights from a cognitive neuroscience perspective. *J. Manag. Inf. Syst.* 31 6–48. 10.1080/07421222.2014.1001255

[B53] Ifinedo P. (2014). Information systems security policy compliance: an empirical study of the effects of socialisation, influence, and cognition. *Inf. Manag.* 51 69–79. 10.1016/j.im.2013.10.001

[B54] Jacobs N. Goossens L. Dehue F. Völlink T. Lechner L. (2015). Dutch cyberbullying victims’ experiences, perceptions, attitudes and motivations related to (coping with) cyberbullying: focus group interviews. *Societies* 5 43–64. 10.3390/soc5010043

[B55] Jagatic T. N. Johnson N. A. Jakobsson M. Menczer F. (2007). Social phishing. *Commun. ACM* 50 94–100.

[B56] Jakobsson M. Ratkiewicz J. (2006). “Designing ethical phishing experiments: a study of (ROT13) rOnl query features,” in *Proceedings of the 15th International Conference on World Wide Web Feature*, Scotland

[B57] Joireman J. Shaffer M. J. Balliet D. Strathman A. (2012). Promotion orientation explains why future-oriented people exercise and eat healthy evidence from the two-factor consideration of future consequences-14 scale. *Pers. Soc. Psychol. Bull.* 38 1272–1287. 10.1177/0146167212449362 22833533

[B58] Jones A. Colwill C. (2008). “Dealing with the Malicious Insider,” in *Proceedings of the 6th Australian Information Security Management Conference*, (Perth,WA: Edith Cowan University).

[B59] Kar K. Moustafa A. A. Myers C. E. Gluck M. A. (2010). “Using an animal learning model of the hippocampus to simulate human fMRI data,” in *Proceedings of the 2010 IEEE 36th Annual Northeast Bioengineering Conference (NEBEC)*, New York, NY.

[B60] Keller U. Strobel A. Wollschläger R. Greiff S. Martin R. Vainikainen M. (2019). A need for cognition scale for children and adolescents: structural analysis and measurement invariance. *Eur. J. Psychol. Assess.* 35 137–149. 10.1027/1015-5759/a000370

[B61] King Z. M. Henshel D. S. Flora L. Cains M. G. Hoffman B. Sample C. (2018). Characterizing and measuring maliciousness for cybersecurity risk assessment. *Front. Psychol.* 9:39. 10.3389/fpsyg.2018.00039 29459838 PMC5807417

[B62] Knott B. A. Mancuso V. F. Bennett K. Finomore V. McNeese M. McKneely J. A. (2013). “Human factors in cyber warfare: alternative perspectives,” in *Proceedings of the Human Factors and Ergonomics Society Annual Meeting*, Los Angeles, CA.

[B63] Landwehr C. (1981). Formal models of computer security. *Comput. Surv.* 13 247–278. 10.1145/356850.356852

[B64] Lee Y. Kozar K. A. (2005). Investigating factors affecting the adoption of anti-spyware systems. *Commun. ACM* 48 72–77. 10.1145/1076211.1076243

[B65] Lin Y. Durbin J. M. Rancer A. S. (2016). Math anxiety, need for cognition, and learning strategies in quantitative communication research methods courses. *Commun. Q.* 64 390–409. 10.1080/01463373.2015.1103294

[B66] Maasberg M. Van Slyke C. Ellis S. Beebe N. (2020). The dark triad and insider threats in cyber security. *Commun. ACM* 63 64–80. 10.1145/3408864

[B67] Mancuso M. Christensen J. C. Cowley J. Finomore V. (2014). “Human factors in cyber warfare II: emerging perspectives,” in *Proceedings of the Human Factors and Ergonomics Society Annual Meeting*, (Thousand Oaks, CA: SAGE Publications), 58.

[B68] Maqbool Z. Aggarwal P. Pammi V. S. C. Dutt V. (2020). Cyber security: effects of penalizing defenders in cyber-security games via experimentation and computational modeling. *Front. Psychol.* 11:11. 10.3389/fpsyg.2020.00011 32063872 PMC6999552

[B69] Maurushat A. (2010). Hackers, Fraudsters and Botnets: Tackling the Problem of Cyber Crime – The Report of the Inquiry into Cyber Crime Invited Submission to the House of Representatives Standing Committee on Communications, Parliament of Australia. Available online at: http://aph.gov.au/house/committee/coms/cybercrime/report/full_report.pdf

[B70] May A. C. Aupperle R. L. Stewart J. L. (2020). Dark times: the role of negative reinforcement in methamphetamine addiction. *Front. Psychiatry* 11:114. 10.3389/fpsyt.2020.00114 32256392 PMC7090143

[B71] McAlaney J. Taylor J. Faily S. (2015). The social psychology of cybersecurity. *Paper presented at the 1st International Conference on Cyber Security for Sustainable Society. Coventry.*

[B72] McBride M. Carter L. Warkentin M. (2012). Exploring the role of individual employee characteristics and personality on employee compliance with cyberse-curity policies. *RTI Int. Inst. Homel. Secur. Solut.* 5:1. 10.1016/j.paid.2019.05.040

[B73] Mishra S. Dhillon G. (2006). “Information systems security governance research: a behavioral perspective,” in *Proceedings of the 1st Annual Symposium on Information Assurance, academic track of the 9th Annual 2006 NYS Cyber Security Conference*, New York, NY.

[B74] Mohebzada J. El Zarka A. BHojani A. H. Darwish A. (2012). “Phishing in a university community: Two large scale phishing experiments,” in *Proceedings of the Innovations in Information Technology (IIT), International Conference*, (Piscataway, NJ: IEEE), 249–254.

[B75] Moustafa A. A. Cohen M. X. Sherman S. J. Frank M. J. (2008). A role for dopamine in temporal decision making and reward maximization in parkinsonism. *J. Neurosci.* 28 12294–12304. 10.1523/jneurosci.3116-08.2008 19020023 PMC3049941

[B76] Moustafa A. A. Keri S. Herzallah M. M. Myers C. E. Gluck M. A. (2010). A neural model of hippocampal-striatal interactions in associative learning and transfer generalization in various neurological and psychiatric patients. *Brain Cogn.* 74 132–144. 10.1016/j.bandc.2010.07.013 20728258 PMC2936107

[B77] Moustafa A. A. Keri S. Polner B. White C. (2017). Drift diffusion model of reward and punishment learning in rare alpha-synuclein gene carriers. *J. Neurogenet.* 31 17–22. 10.1080/01677063.2017.1301939 28316265

[B78] Moustafa A. A. Keri S. Somlai Z. Balsdon T. Frydecka D. Misiak B. (2015). Drift diffusion model of reward and punishment learning in schizophrenia: modeling and experimental data. *Behav. Brain Res.* 291 147–154. 10.1016/j.bbr.2015.05.024 26005124

[B79] Moustafa A. A. Krishna R. Eissa A. M. Hewedi D. H. (2013). Factors underlying probabilistic and deterministic stimulus-response learning performance in medicated and unmedicated patients with Parkinson’s disease. *Neuropsychology* 27 498–510. 10.1037/a0032757 23876122

[B80] Moustafa A. A. Morris A. N. ElHaj M. (2018a). A review on future episodic thinking in mood and anxiety disorders. *Rev. Neurosci.* 30 85–94. 10.1515/revneuro-2017-0055 29858910

[B81] Moustafa A. A. Morris A. N. Nandrino J. L. Misiak B. Szewczuk-Boguslawska M. Frydecka D. (2018b). Not all drugs are created equal: impaired future thinking in opiate, but not alcohol, users. *Exp. Brain. Res.* 236 2971–2981. 10.1007/s00221-018-5355-7 30099573

[B82] Moustafa A. A. Myers C. E. Gluck M. A. (2009). A neurocomputational model of classical conditioning phenomena: a putative role for the hippocampal region in associative learning. *Brain Res.* 1276 180–195. 10.1016/j.brainres.2009.04.020 19379717

[B83] Moutsiana C. Garrett N. Clarke R. C. Lotto R. B. Blakemore S. J. Sharot T. (2013). Human development of the ability to learn from bad news. *Proc. Natl. Acad. Sci. U.S.A.* 110 16396–16401. 10.1073/pnas.1305631110 24019466 PMC3799330

[B84] Myers C. E. Sheynin J. Balsdon T. Luzardo A. Beck K. D. Hogarth L. (2016). Probabilistic reward- and punishment-based learning in opioid addiction: experimental and computational data. *Behav. Brain Res.* 296 240–248. 10.1016/j.bbr.2015.09.018 26381438 PMC4734141

[B85] Nobles C. (2018). Botching human factors in cybersecurity in business organizations. *Holistica* 9 71–88. 10.2478/hjbpa-2018-0024

[B86] Parsons K. Calic D. Pattinson M. Butavicius M. McCormaca A. Zwaans T. (2017). The human aspects of information security questionnaire (HAIS-Q): two further validation studies. *Comput. Secur.* 55 40–51. 10.1016/j.cose.2017.01.004

[B87] Patton J. H. Stanford M. S. Barratt E. S. (1995). Factor structure of the barratt impulsiveness scale. *J. Clin. Psychol.* 51 768–774. 10.1002/1097-4679(199511)51:6<768::aid-jclp2270510607>3.0.co;2-1 8778124

[B88] Paulhus D. Williams K. (2002). The dark triad of personality: narcissism, machiavellianism, and psychopathy. *J. Res. Pers.* 36 556–563. 10.1016/s0092-6566(02)00505-6

[B89] Peckham A. D. Johnson S. L. (2018). Cognitive control training for emotion-related impulsivity. *Behav. Res. Ther.* 105 17–26. 10.1016/j.brat.2018.03.009 29609103 PMC5937944

[B90] Piray P. Zeighami Y. Bahrami F. Eissa A. M. Hewedi D. H. Moustafa A. A. (2014). Impulse control disorders in Parkinson’s disease are associated with dysfunction in stimulus valuation but not action valuation. *J. Neurosci.* 34 7814–7824. 10.1523/jneurosci.4063-13.2014 24899705 PMC6608260

[B91] Rajivan P. Aharonov-Majar E. Gonzalez C. (2020). Update now or later? Effects of experience, cost, and risk preference on update decisions. *J. Cyber Secur.* 6:tyaa002.

[B92] Rajivan P. Gonzalez C. (2018). Creative persuasion: a study on adversarial behaviors and strategies in phishing attacks. *Front. Psychol.* 9:135. 10.3389/fpsyg.2018.00135 29515478 PMC5826381

[B93] Rankin C. H. Abrams T. Barry R. J. Bhatnagar S. Clayton D. F. Colombo J. (2009). Habituation revisited: an updated and revised description of the behavioral characteristics of habituation. *Neurobiol. Learn. Mem.* 92 135–138. 10.1016/j.nlm.2008.09.012 18854219 PMC2754195

[B94] Regier P. S. Redish A. D. (2015). Contingency management and deliberative decision-making processes. *Front. Psychiatry* 6:76. 10.3389/fpsyt.2015.00076 26082725 PMC4450586

[B95] Rodriguez-Enriquez M. Bennasar-Veny M. Leiva A. Garaigordobil M. Yanez A. M. (2019). Cybervictimization among secondary students: social networking time, personality traits and parental education. *BMC Public Health* 19:1499. 10.1186/s12889-019-7876-9 31711467 PMC6849165

[B96] Rosenbaum G. M. Botdorf M. A. Patrianakos J. L. Steinberg L. Chein J. M. (2017). Working memory training in adolescents decreases laboratory risk taking in the presence of peers. *J. Cogn. Enhanc.* 1 513–525. 10.1007/s41465-017-0045-0 29457149 PMC5813817

[B97] Sadkhan S. B. (2019). Cognition and the future of information security. *Paper presented at the 2019 International Conference on Advanced Science and Engineering (ICOASE).*

[B98] Saleme D. Moustafa A. A. (2020). “The multifaceted nature of risk-taking in drug addiction,” in *Cognitive, Clinical, and Neural Aspects of Drug Addiction*, ed. Moustafa A. A. (Amsterdam: Elsevier).

[B99] Saleme D. M. Kluwe-Schiavon B. Soliman A. Misiak B. Frydecka D. Moustafa A. A. (2018). Factors underlying risk taking in heroin-dependent individuals: Feedback processing and environmental contingencies. *Behav. Brain Res.* 350 23–30. 10.1016/j.bbr.2018.04.052 29778626

[B100] Sandouka H. Cullen A. Mann I. (2009). Social engineering detection using neural networks. *Paper Presented at the International Conference on CyberWorlds.*

[B101] Sarmany-Schuller I. (1999). Procrastination, need for cognition and sensation seeking. *Stud. Psychol.* 41 73–85.

[B102] Sasse M. A. Brostoff S. Weirich D. (2004). Transforming the weakest link – a human/computer interaction approach to usable and effective security. *BT Technol. J.* 19 122–131.

[B103] Schechter S. Dhamija R. Ozment A. Fischer I. (2007). “The emperor’s new security indicators,” in *Proceedings of the IEEE Symposium on Security and Privacy*, Berkeley, CA.

[B104] Schneier B. (2004). *Secrets and Lies: Digital Security in a Networked World.* Hoboken, NJ: Wiley.

[B105] Scott S. G. Bruce R. A. (1995). Decision-making style: the development and assessment of a new measure. *Educ. Psychol. meas.* 55 818–831. 10.1177/0013164495055005017

[B106] Sharot T. (2011). The optimism bias. *Curr. Biol.* 21 R941–R945. 10.1016/j.cub.2011.10.030 22153158

[B107] Shropshire J. Warkentin M. Johnston A. C. Schmidt M. B. (2006). Personality and IT security: an application of the five-factor model. *Paper presented at the Connecting the Americas, 12th Americas Conference on Information Systems*, (Acapulco: AMCIS).

[B108] Sidman M. (2006). The distinction between positive and negative reinforcement: some additional considerations. *Behav. Anal.* 29 135–139. 10.1007/bf03392126 22478460 PMC2223177

[B109] Smith S. W. (2003). Humans in the loop human–computer interaction and security. *IEEE Comput. Soc.* 1 75–79. 10.1109/msecp.2003.1203228

[B110] Stanton J. M. Stam J. R. Mastrangelo P. M. Jolton J. A. (2005). Analysis of end user security behaviors. *Comput. Secur.* 24 124–133. 10.1016/j.cose.2004.07.001

[B111] Thompson P. (2004). Cognitive hacking and intelligence and security informatics. *Proc. SPIE* 5423 142–151.

[B112] Tversky A. Kahneman D. (1986). Rational choice and the framing of decisions. *J. Bus.* 59 251–278.

[B113] Tyworth M. Giacobe N. A. Mancuso V. F. McNeese M. D. Hall D. L. (2013). A human-in-the-loop approach to understanding situation awareness in cyber defence analysis. *EAI Endorsed Trans. Secur. Safe.* 13:e6. 10.4108/trans.sesa.01-06.2013.e6

[B114] Veksler V. D. Buchler N. Hoffman B. E. Cassenti D. N. Sample C. Sugrim S. (2018). Simulations in cyber-security: a review of cognitive modeling of network attackers, defenders, and users. *Front. Psychol.* 9:691. 10.3389/fpsyg.2018.00691 29867661 PMC5967149

[B115] Veksler V. D. Buchler N. LaFleur C. G. Yu M. S. Lebiere C. Gonzalez C. (2020). Cognitive models in cybersecurity: learning from expert analysts and predicting attacker behavior. *Front. Psychol.* 11:1049. 10.3389/fpsyg.2020.01049 32612551 PMC7308471

[B116] Vroom C. von Solms R. (2004). Towards information security behavioural compliance. *Comput. Secur.* 23 191–198. 10.1016/j.cose.2004.01.012

[B117] West R. (2008). The psychology of security: why do good users make bad decisions. *Commun. ACM* 51 34–40.

[B118] Whitty M. Doodson J. Creese S. Hodges D. (2015). Individual differences in cyber security behaviors: an examination of who is sharing passwords. *Cyberpsychol. Behav. Soc. Netw.* 18 3–7. 10.1089/cyber.2014.0179 25517697 PMC4291202

[B119] Wiederhold B. K. (2014). The role of psychology in enhancing cybersecurity. *Cyberpsychol. Behav. Soc. Netw.* 17 131–132. 10.1089/cyber.2014.1502 24592869

[B120] Wogalter M. S. (2006). “Communication-human information processing (C-HIP) model,” in *Handbook of Warnings*, ed. Wogalter M. S. (Mahwah, N.J: Erlbaum), 51–61.

